# Transcriptomic Profiling of Various Developmental Stages of *Aphis Aurantii* to Provide a Genetic Resource for Gene Expression and SSR Analysis

**DOI:** 10.3389/fphys.2020.578939

**Published:** 2020-09-18

**Authors:** Feng Hong, Si-Hua Mo, Yinghong Liu, Dong Wei

**Affiliations:** ^1^College of Agriculture, Xinyang Agriculture and Forestry University, Xinyang, China; ^2^Chongqing Key Laboratory of Entomology and Pest Control Engineering, College of Plant Protection, Southwest University, Chongqing, China; ^3^State Cultivation Base of Crop Stress Biology for Southern Mountainous Land, Academy of Agricultural Sciences, Southwest University, Chongqing, China

**Keywords:** tea aphid, transcriptome, molting, wing dimorphism, simple sequence repeats

## Introduction

The tea aphid, *Aphis aurantii* Boyer de Fonscolombe (Hemiptera: Aphididae), is found in regions where the tea plant grows, and has become one of the most important pests in tea gardens in the tropics and subtropics (Han et al., 2012; Deng et al., 2019). This species is also known as the black citrus aphid and destroys citrus orchards (Wang and Tsai, 2001). *Ap. aurantii* is a polyphagous aphid with over 190 genera hosts, including many other economically important plants in addition to tea and citrus, such as coffee, cacao, loquat, litchi, mango, and camellia (Carver, 1978; Deng et al., 2019). This aphid directly damages trees by sucking the phloem sap out of the shoot tip or from new fresh leaves and injecting its saliva, which causes phytotoxicity and stunting in the plant (Guidolin and Consoli, 2018). Moreover, this aphid secretes honeydew when sap-feeding, and sooty molds frequently grow on the honeydew, which hinders photosynthetic activity (Sevim et al., 2012).

Aphids are wide-spread pests that feed on a wide range of fruits and vegetables, most of them are vectors of plant viruses (Huang and Qiao, 2014; Hulle et al., 2020). Except the tea aphid, there have been previous functional studies on genes associated with the development (Ding et al., 2017; Ye et al., 2019), reproduction (Shang et al., 2018; Ullah et al., 2019b), wing development (Shang et al., 2020b), response to the stress (Gao et al., 2018; Jing et al., 2018), and pest control (Mohammed et al., 2018; Ullah et al., 2019a) of other aphids pests (e.g., *Ap. citricidus, Ap. gossypii, Acyrthosiphon pisum*). Moreover, functional studies of the new insecticide targets in aphids were also conducted in these aphids (Ye et al., 2019; Shang et al., 2020a). However, most previously published papers have focused on the ecology (Alizadeh Kafeshani et al., 2018; Guidolin and Consoli, 2018; Li et al., 2019), control (Aslam et al., 2015; Gholamzadeh-Chitgar and Pourmoradi, 2017), and mitochondrial genome (Wang et al., 2019) in tea aphid *Ap. aurantii*. There have been few functional studies that focused on the development and reproduction of *Ap. aurantii*, because there are limited reference sequences.

Quantitative and qualitative transcriptome analyses can reveal the integrated biochemical and physiological processes at a molecular level that are associated with specific aspects of the organism, such as identification of the critical genes during the different developmental stages in insects (Morandin et al., 2018; Liu et al., 2020). For instance, RNA-Seq was used to elucidate the underlying molecular mechanisms of metamorphic development of *Henosepilachna vigintioctopunctata* (Zhang et al., 2018). Using available insect genomes, comparative transcriptome analysis was conducted to analyze gene expression during all developmental stages of *Zeugodacus cucurbitae* (Wei et al., 2020), *Bactrocera dorsalis* (Liu et al., 2020). In addition, gene expression has been studied by RNA-Seq in multiple tissues to identify tissue-specific genes involved in female fertility in *B. dorsalis* [e.g., vitellogenin and vitelline membrane protein in female (Wei et al., 2018, 2019)]. In aphids, RNA-Seq was used to analyze the gene expression between dispersing and non-dispersing morphs (Shang et al., 2016, 2020b). Thus, RNA-Seq technology allows us to determine the gene expression to underlie certain biological functions of critical genes and identify potential targets of new environmentally friendly insecticides for pest control.

Simple sequence repeats (SSRs), also known as microsatellites, are short, tandemly arranged, repeating motifs (1–6 bp), which are widely distributed throughout the genomes of eukaryotic organisms (Temnykh et al., 2001). SSRs are co-dominant, hypervariable, neutral, and reproducible molecular markers; therefore, they have become the most widely used molecular markers in population genetic and conservation studies to evaluate the level of genetic variation in a species (King, 2012). Transcriptomic sequencing is also a highly efficient approach to identify SSRs in insects with no accessible genome. This method was extensively used to identify and analyze the SSRs in *Liposcelis entomophila* (Wei et al., 2013) and *H. vigintioctopunctata* (Zhang et al., 2018). SSRs were also identified in specific tissues in *B. dorsalis* (Wei et al., 2015).

In this study, RNA-Seq was conducted on samples from six stages of *Ap. aurantii* with four biological replicates. A comprehensive transcriptome was sequenced, the transcripts were *de novo* assembled, and the gene's functional annotation was performed. Gene expression during development and SSRs were analyzed. These results will be valuable for the future functional studies of genes involved in *Ap. aurantii* development, reproduction, and wing differentiation.

## Materials and Methods

### Insects

The aphid *Ap. aurantii* was collected as wingless adults from a tea orchard in Xinyang, Henan Province, China, 2019. Aphids were reared in the laboratory at 25°C with a relative humidity of 75% and photoperiod of 14: 10 h light: dark (Wang and Tsai, 2001). All adults and nymphs were reared with fresh young tender tea shoots. The density of aphids was controlled, and the nymphs and wingless adults can be easily collected from the tea shoots. To obtain the winged aphids, the density of aphid in each tea shoot was not controlled and the high density induced the wing dimorphism. The winged aphids were moved to a new tea shoot for 1 day feeding before sampling.

### Sample Preparation and Total RNA Isolation

Newly emerged nymphs (<4 h) were collected and cultured separately. The 1st instar nymphs (~24 h, 100 individuals), 2nd instar nymphs (3-d-old, 70 individuals), 3rd instar nymphs (5-d-old, 30 individuals), wingless 4th instar nymphs (6-d-old, 20 individuals), and wingless and winged adults (7d-old, 20 individuals) were collected per replicate. Four biological replicates were collected at each stage. Total RNA was isolated from each sample using TRIzol reagent (Invitrogen, Carlsbad, CA, USA) following the manufacturer's protocol. RNA concentration was measured using a Qubit RNA Assay Kit in a Qubit® 2.0 Flurometer (Life Technologies, Carlsbad, CA, USA). RNA integrity was evaluated by agarose gel electrophoresis, and then assessed using the Agilent Bioanalyzer 2100 system (Agilent Technologies, Palo Alto, CA, USA).

### Library Construction and Transcriptomic Sequencing

The transcriptome libraries were constructed using the NEBNext Ultra™ RNA Library Prep Kit for Illumina (NEB, San Diego, CA, USA) following the standard manufacturer's instructions. In brief, 1.5 μg total RNA in each sample was used for mRNA enrichment using poly-T oligo-attached magnetic beads. Fragmentation was conducted using divalent cations under elevated temperature in NEBNext First Strand Synthesis Reaction Buffer. Then, cDNA was synthesized using the NEBNext First-Strand Synthesis kit. Remaining overhangs were converted into blunt ends via exonuclease/polymerase activities. After adenylation of 3′ ends of DNA fragments, NEBNext Adaptor with hairpin loop structure was ligated to prepare for hybridization. For the size selection of fragments (250–300 bp), 3 μl USER Enzyme (NEB, USA) was used with size-selected, adaptor-ligated cDNA at 37°C for 15 min followed by 95°C for 5 min. Then, PCR amplification was performed with Phusion High-Fidelity DNA polymerase (Thermo Scientific), Universal PCR primers, and Index (X) Primer. Finally, the PCR products were purified (AMPure XP system) and the quality was re-assessed on the Agilent Bioanalyzer 2100 system.

### Sequencing Processing and Assembly

After cluster generation on a cBot Cluster Generation System using TruSeq PE Cluster Kit v3cBot-HS (Illumia), the library preparations were sequenced by Novogene Co. Ltd. and generated paired-end reads (2 × 150-bp read length). Raw data were first processed through in-house perl scripts to remove the adapter, low-quality reads, and reads containing poly N. The error rate of each read was determined by the Phred score. In this step, clean reads were obtained from the clean data, and Phred score >20 (Q20), GC content, and sequence duplication level of the clean data were evaluated. All the following analyses were based on these high-quality clean data. The *de novo* assembly was accomplished based on the clean reads using Trinity v2.4.0 with default parameters (Grabherr et al., 2011).

### Functional Annotation

Gene function was annotated based on the following seven databases: National Center of Biotechnology Information (NCBI) non-redundant protein sequences (NR), a manually annotated and reviewed protein sequence database (SwissProt), Protein family (Pfam), NCBI non-redundant nucleotide sequences (NT), euKaryotic Ortholog Groups (KOG), Gene Ontology (GO), and Kyoto Encyclopedia of Genes and Genomes (KEGG). First, the proteins of identified genes were predicted by BLASTx against the NCBI NR and SwissProt databases using Diamond v0.8.22 with an *E*-value <10^−5^. The functional annotations for proteins with the highest sequence similarity to the given genes were retrieved. Gene sequences were then aligned to the Pfam database using HMMER v3.0 with an *E*-value < 0.01, and then aligned to the NCBI NT database by BLASTn with an *E*-value < 10^−5^. KOG is a database in which orthologous gene products are classified. Genes were aligned to the KOG database using Diamond with an *E*-value < 10^−5^ to predict and classify the possible functions of the genes. The functional annotations were all determined based on the highest sequence similarity in these databases. For GO annotation, blast2GO v2.5 was used to obtain GO terms with an *E*-value < 10^−5^ (Stefan et al., 2008). The biochemical pathway information was analyzed by downloading relevant maps from the KEGG database (Kanehisa and Goto, 2000), a database that contains a comprehensive analysis of inner-cell metabolic pathways and functions of each gene product. We used KOBAS 2.0 to test the statistical enrichment in KEGG pathways (Xie et al., 2011). These pathways are useful for studying complex biological behaviors. In this study, KEGG pathway analysis was performed using the KEGG automatic annotation server with an *E*-value < 10^−10^.

### Gene Expression During Development

Gene expression levels were inferred using RSEM v1.2.15 (Li and Dewey, 2011) with the fragments per kilobase of exon model per million mapped reads (FPKM) method (Florea et al., 2013). Clean data for each sample were mapped back onto the assembled transcriptome, and the read count for each gene was obtained from the mapping results. The FPKM values of the biological replicates were analyzed by Pearson correlation. Only an average FPKM value > 0.3 in the four replicates was considered expressed during the given stage. In this study, differential expression analysis of two related developmental stages was performed using the DESeq2 method by comparing the normalized read count (Love et al., 2014). The resulting *P*-values were adjusted using the Benjamini and Hochberg approach to control the false discovery rate (Benjamini and Hochberg, 1995). Those genes with a two-fold difference between two stages and an adjusted *P* < 0.05 were identified as differentially expressed genes (DEGs).

### SSR Discovery

To facilitate inheritance studies and evaluate the assembly quality, we analyzed the SSR distribution in tea aphids using MISA v1.0 (http://pgrc.ipk-gatersleben.de/misa/misa.html) with the default parameters (Sharma et al., 2007).

## Results and Discussion

### Data Description and Processing

In this study, a total of 1.19 G raw reads was obtained by sequencing, and 1.15 G clean reads with 173.13 G clean bases were filtered for the assembly (Table S1). The error rate of each read was 0.03% in all samples. The mean Q20 was 97.54. The GC content in all samples ranged from 33.83–37.65%. The GC contents differed among insects. For example, there was ~43% GC content in the transcriptomes of *Z. cucurbitae* (Wei et al., 2020) and *B. dorsalis* (Liu et al., 2020). The GC content in *Ap. aurantii* (35.66%) was also lower than that of another aphid *Ap. citricidus* (41.97%) (Shang et al., 2016). After *de novo* assembly based on the clean reads, a total of 176,161 transcripts of 72,868 genes were assembled. The mean length of these transcripts and genes were 1,229 and 611 bp, respectively, and the N50 values were 4,311 and 2,540, respectively. Length distribution revealed that 38.35 and 16.35% of the fragments were longer than 2,000 bp, respectively.

A clear understanding of the molecular mechanisms that regulate pest life cycles and development at each stage may aid in their control by facilitating the development of more sustainable and environmentally friendly approaches. Transcriptome analyses of several insect species across developmental stages have been reported in many insect species, such as *Acheta domesticus* (Oppert et al., 2020), *Dendrolimus houi* (Han et al., 2019), *Cylas formicarius* (Ma et al., 2016), and *Dr. melanogaster* (Graveley et al., 2011). In this study, we described a draft transcriptome from various developmental stages of the tea aphid, which substantially increases the molecular resources available for elucidating gene expression during insect development and functional study of development and reproduction.

### Functional Annotation Results

All distinct sequences were annotated against seven databases, and a total of 47,825 distinct genes (65.63%) were annotated, which means that there is still a gap in knowledge regarding the genetic information of this species. In total, 38,268 genes (52.51%) matched known genes that encoded functional proteins in the NCBI NR database, and 41,474, 29,095, 30,133, 14,160, 30,133, and 6,378 genes were annotated to the NCBI NT, SwissProt, Pfam, KOG, GO, and KEGG databases, respectively. Among these genes, only 3,971 genes (5.44%) were annotated in all seven databases. Almost one-third shared no significant similarities to known genes/proteins and may be novel or fast-evolving sequences. These sequences may have a role in host selection and speciation, and represent ideal subjects for future evolutionary genetic studies on this species.

In the KOG analysis, 14,160 genes were annotated into 26 groups, in which four groups had > 1,000 genes (Figure S1A). The most prominent group was “general function prediction only” (1,884 genes), followed by “posttranslational modification, protein turnover, chaperones” (1,538 genes), “signal transduction mechanisms” (1,389 genes), and “translation, ribosomal structure, and biogenesis” (1,228 genes). GO analysis incudes three categories (biological process, cellular component, molecular function). All of these 30,133 genes were thereafter assigned into 56 terms (Figure S1B). Specifically, in the biological process category, “cellular process” (17,575 genes), “metabolic process” (16,182 genes), and “single-organism process” (14,605 genes) were the top three terms. In the cellular component category, “cell and cell part” (9,049 genes) was the most dominant term. In the molecular function category, “binding” (15,344 genes) and “catalytic activity” (13,869 genes) were two most dominant terms. The distribution of genes in COG and GO databases were similar to the *Ap. citricidus* results (Shang et al., 2016). In the insects for which genomes are available, the proportion of annotated genes was much higher (e.g., 75.4% of genes were annotated in *Z. cucurbitae*) (Wei et al., 2020).

In KEGG pathway analysis, a total of 6,378 unigenes were annotated into 229 pathways which contributed to five level 1 and 32 level 2 pathway hierarchies (Figure S2). In the organismal systems hierarchy, 72, 236, 259, and 71 unigenes were involved in development, digestion, immunity, and sense, respectively. In the metabolism hierarchy, 414, 503, and 297 were involved in amino acid metabolism, carbohydrate metabolism, and lipid metabolism, respectively. In other hierarchies, translation (477), signal transduction (667), and transport and catabolism (401) were the most dominant terms in genetic information processing, environmental information processing, and cellular processes, respectively.

### Gene Expression During Development

**Figure 1:**
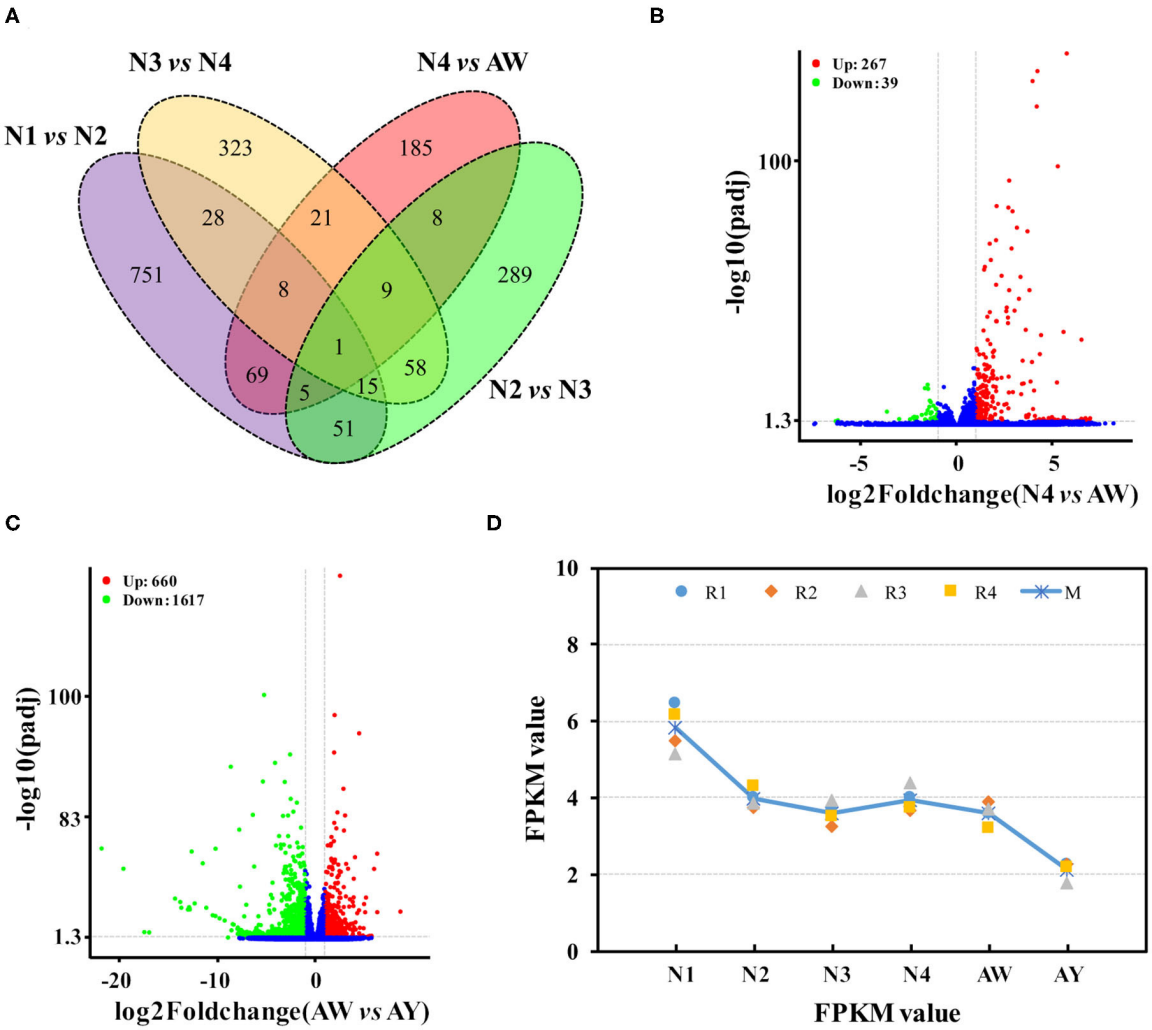
Gene expression during developmental stages in *Aphis aurantii*. **(A)** Venn diagram of differentially expressed genes during developmental stages of wingless aphids. **(B)** Volcano plot of gene expression between wingless 4th instar nymphs and wingless adults. **(C)** Volcano plot of gene expression between wingless and winged adults. **(D)** An example of gene expression in all stages. N1, N2, N3, and N4 indicates the 1st-, 2nd-, 3rd-, 4th-instar nymph respectively. AW and AY indicates wingless and winged adults respectively.

Gene expression data revealed dynamic changes among different stages. A total of 53,258 genes were qualitatively expressed in at least one stage. Comparison of gene expression in successive stages revealed that 3,708 genes were differentially expressed in at least one ecdysis process or between the wingless and winged adults. Of these genes, 928, 436, and 463 were differentially expressed between 1st, 2nd, and 3rd molting process respectively, and 306 genes were differentially expressed in the last ecdysis (Figure 1A). These DEGs were most likely to be involved in ecdysis development. For example, of the 306 DEGs between 4th instar nymphs and wingless adults (Figure 1B), 267 unigenes were up-regulated in wingless adults, and 39 genes were down-regulated. Among the four ecdysis processes, 1,821 DEGs were screened out (Table S2). Gene expression is very important for understanding gene function during developmental stages. The DEGs play critical roles during the ecdysis development, and are potential targets of new environmentally friendly insecticides. In this study, only 16 genes were dynamically expressed in the first three molting stages, and 306 genes showed differential expression in the last ecdysis stage from wingless 4th instar nymphs to wingless adults; alternatively, only one gene differed among all ecdysis stages.

In addition, 2,277 DEGs were found between the wingless and winged adults (Table S3). Of these unigenes, 660 were highly expressed in wingless adults, and 1,617 unigenes were highly expressed in winged adults (Figure 1C). It is reasonable that there are more differences in gene expression because of their morphological differences. Wing dimorphism is a phenomenon of phenotypic plasticity associated with aphid dispersal. Aphids have been demonstrated to have high phenotypic plasticity and can transform from wingless to winged forms under conditions of over-crowding, lack of nutrition, and interactions with other organisms (Shang et al., 2020b). Diverse processes take place during wing dimorphism and development (Zhang et al., 2019). Many studies focused on the regulatory mechanism of wing dimorphism and development (Xu et al., 2015; Song et al., 2018; Shang et al., 2020b). Finally, 390 of these DEGs also showed differences in the last ecdysis process from 4th instar nymph to wingless adult, and 1,887 genes only showed differences between winged and wingless adults. Alternatively, many more genes showed differential expression in *A. citricidus* (Shang et al., 2016). In addition to the DEG analysis, quantitative transcriptomic analysis also provided basic information regarding gene expression of all assembled genes in *Ap. aurantii* [e.g., an example of *CYP315A1* gene (*Cluster26641*) (Figure 1D)].

### SSR Identification

**Figure 2:**
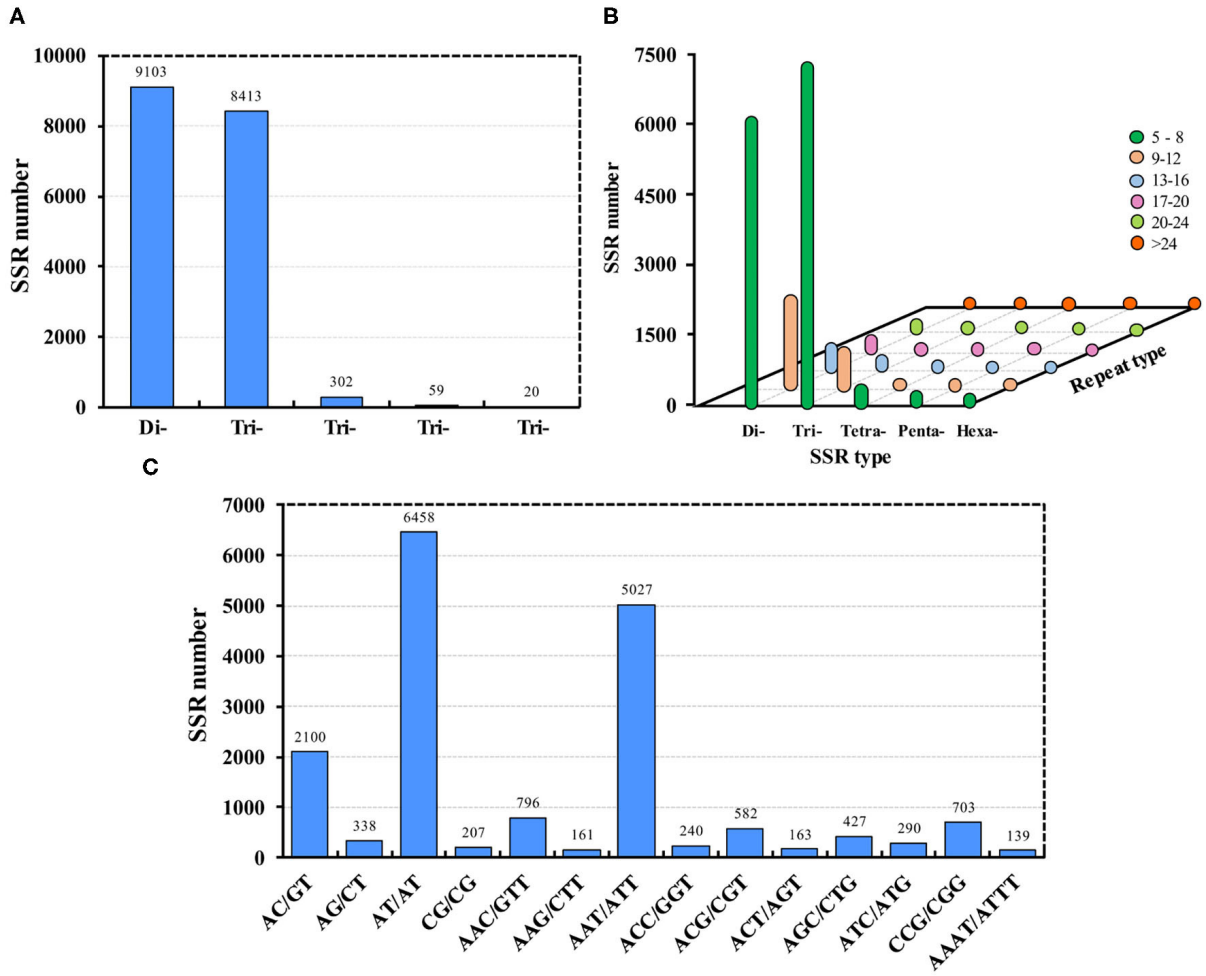
SSRs identified in *Aphis aurantii*. **(A)** Number of SSRs of different types. **(B)** SSR repeats of each type. **(C)** The top 14 SSRs, of which there were more than 100 each.

In total, 22,847 genes (31.35%) contained SSRs; 10,550 genes contained more than one SSR. By removing the mono-nucleotide repeats, a total of 17,897 SSRs were identified, of which 9,103 were di-nucleotide repeats and 8,413 were tri-nucleotide repeats (Figure 2A). Most of the SSRs were 5–8 repeats (Figure 2B). Additionally, the most popular SSRs were AT/TA, AAT/ATT, and AC/GT, of which there were more than 2,000 each (Figure 2C). Recently, 4,138 SSRs were identified in all developmental stages of *De. houi* using the same RNA-Seq technology (Han et al., 2019). Similar to SSRs, single nucleotide polymorphisms (SNPs) are also relevant in development and reproduction; they are usually considered point mutations that occur among alleles at a locus, and can be readily identified from assemblies using computational methods. The transcriptome produced in this study provides a valuable resource for SNP identification in *Ap. aurantii*.

## Data Availability Statement

All of the sequenced raw data were deposited into the NCBI Sequence Read Archive (SRA) database under accession number PRJNA610500. The accession number of each running experiment were SRR11241577, SRR11241578, SRR11241579, SRR11241580, SRR11241581, SRR11241582, SRR11241583, SRR11241584, SRR11241585, SRR11241586, SRR11241587, SRR11241588, SRR11241589, SRR11241590, SRR11241591, SRR11241592, SRR11241593, SRR11241594, SRR11241595, SRR11241596, SRR11241597, SRR11241598, SRR11241599, SRR11241600, respectively.

## Author Contributions

DW and FH conceived, designed the study, and drafted the manuscript. S-HM and YL contributed to the materials and samples. FH and S-HM analyzed the data and contributed to the tables and figures. All authors revised the manuscript and approved the final manuscript.

## Conflict of Interest

The authors declare that the research was conducted in the absence of any commercial or financial relationships that could be construed as a potential conflict of interest.
