# Supplementary material for: Transcriptomic Profiling of Various Developmental Stages of Aphis Aurantii to Provide a Genetic Resource for Gene Expression and SSR Analysis
Source: Front Physiol. 2020 Sep 18;11:578939. doi: 10.3389/fphys.2020.578939 (PMC7530277; doi:10.3389/fphys.2020.578939)
Supplement: Supplementary file 1 [file Table_1.docx]

Supplementary Figures

**Figure S1.** EuKaryotic Ortholog Groups (KOG) classification of genes in *Aphis aurantii* (**A**), gene ontology classification of genes in *Aphis aurantii* (**B**).

**Figure S2.** Levels one and two pathway analysis of genes in *Aphis aurantii* by mapping to the Kyoto Encyclopedia of Genes and Genomes (KEGG) database.
